# Cell Models and Omics Techniques for the Study of Nonalcoholic Fatty Liver Disease: Focusing on Stem Cell-Derived Cell Models

**DOI:** 10.3390/antiox11010086

**Published:** 2021-12-30

**Authors:** María Pelechá, Estela Villanueva-Bádenas, Enrique Timor-López, María Teresa Donato, Laia Tolosa

**Affiliations:** 1 Unidad de Hepatología Experimental, Instituto de Investigación Sanitaria La Fe, 46026 Valencia, Spain; maria_pelecha@iislafe.es (M.P.); estela.villanueva@uv.es (E.V.-B.); enrique.timor@uv.es (E.T.-L.); 2 Departamento de Bioquímica y Biología Molecular, Facultad de Medicina y Odontología, Universidad de Valencia, 46010 Valencia, Spain; 3 Centro de Investigación Biomédica en Red de Enfermedades Hepáticas y Digestivas (CIBERehd), Instituto de Salud Carlos III, 28029 Madrid, Spain; 4 Biomedical Research Networking Center on Bioengineering, Biomaterials and Nanomedicine (CIBER-BBN), Instituto de Salud Carlos III, 28029 Madrid, Spain

**Keywords:** fatty liver, in vitro models, mechanisms, pluripotent stem cells

## Abstract

Nonalcoholic fatty liver disease (NAFLD) is now the leading cause of chronic liver disease in western countries. The molecular mechanisms leading to NAFLD are only partially understood, and effective therapeutic interventions are clearly needed. Therefore, preclinical research is required to improve knowledge about NAFLD physiopathology and to identify new therapeutic targets. Primary human hepatocytes, human hepatic cell lines, and human stem cell-derived hepatocyte-like cells exhibit different hepatic phenotypes and have been widely used for studying NAFLD pathogenesis. In this paper, apart from employing the different in vitro cell models for the in vitro assessment of NAFLD, we also reviewed other approaches (metabolomics, transcriptomics, and high-content screening). We aimed to summarize the characteristics of different cell types and methods and to discuss their major advantages and disadvantages for NAFLD modeling.

## 1. Introduction

Nonalcoholic fatty liver disease (NAFLD) is the most common liver disorder in western countries, affecting 17–46% of adults and characterized by increased fat accumulation [1]. NAFLD comprises two different histological forms: nonalcoholic fatty liver (or simple steatosis) and nonalcoholic steatohepatitis (NASH) [2]. Steatosis can be defined as the presence of increased fat accumulation in the liver without hepatocellular necrosis and no, or minimal, inflammation. NASH is characterized by steatosis, liver inflammation, and hepatocyte ballooning with or without fibrosis. The progression of NASH underlies cirrhosis and hepatocellular carcinoma [2].

Evidence for hepatic steatosis in the absence of other causes of liver fat accumulation (i.e., significant alcohol consumption or hereditary disorders) is required to diagnose NAFLD, which is often asymptomatic and usually discovered during routine laboratory examinations with high transaminase levels. Most of these patients may have normal ALT levels, which tend to remain low as the disease progresses [3]. NAFLD is characterized by the presence of steatosis in >5% of the hepatocytes assessed by either histological analysis or a proton density fat fraction, determined by proton magnetic resonance imaging or magnetic resonance imaging (MRI) [2]. However, a multicenter study with adults demonstrated that MRI proton density fat fraction showed greater practicality and narrower sampling variability [4,5]. Additionally, European NAFLD management guidelines recommend ultrasonography to diagnose simple steatosis, but predictive serum biomarkers of disease risk and progression are urgently needed [3]. In fact, the international consortia LITMUS, by the European Union, and NIMBLE, by the United States of America, focus on finding reliable NAFLD and NASH biomarkers [6]. The definitive NASH diagnosis and its differentiation from simple steatosis always requires liver biopsy [2].

NAFLD is the result of multiple molecular and cellular changes and involves genetic, dietary, metabolic, and immunological factors [7]. The most common risk factors for NAFLD include obesity, type 2 diabetes, and metabolic syndrome features [8]. Hence, global NAFLD prevalence is expected to increase in forthcoming years in parallel to a rise in insulin resistance and obesity [8], which spells a growing economic challenge. Furthermore, it is widely accepted that there are evident sex differences in NAFLD [9]. The prevalence of the disease and its fibrotic progression follow a sexually dimorphic pattern [10]. Fertile women have been found to be at lower risk of NAFLD than men [11], but post-menopausal women lose the protection conferred by estrogens [9,10,11].

Drug-induced steatosis has the potential to directly produce NAFLD and, although drugs are not normally a primary cause of NAFLD, it has been shown that they may underlie, mimic, or aggravate NAFLD [12]. There are reports that drugs often used in clinical practice, such as amiodarone, methotrexate, tamoxifen, and some chemotherapeutic agents, may lead to hepatic steatosis [12,13].

Although NAFLD is the most common liver disease, no pharmacological treatment is presently approved, and the first line of treatment is lifestyle modification [14]. Even so, more than 200 active trials of NAFLD treatments are presently underway [15]. The multidimensional NAFLD etiopathogenesis is also reflected in drug development, as treatments focus on controlling the different pathways implicated in disease progression, such as chronic inflammation, insulin resistance, and fibrogenesis [7].

The need for safe, effective treatments has urged the development of both in vivo and in vitro models of NAFLD to better understand its pathogenesis, to identify potential targets, and to assess the therapeutic potential of drugs before transferring them to clinics [16]. In vitro models of NAFLD comprise simple cell-based models incubated with different lipids to more complex three-dimensional (3D) organoids. These liver cell-based NAFLD models include conventionally cultured hepatic cell lines, primary human hepatocytes (PHH), cocultures of different liver cells, and engineered liver platforms, such as perfusion systems [17]. Disease heterogeneity is relevant in clinical practice for using more personalized diagnostic and treatment approaches [18], and also in the preclinical setting to utilize in vitro models that better reflect population variability (including sex differences in NAFLD and differential drug responses). To achieve more patient-specific human cell-based models that accurately mimic pathologies in the liver, new in vitro systems have recently been developed, of which induced pluripotent stem cells (iPSCs) differentiated into hepatocyte-like cells (HLCs) are a promising option for liver disease modeling in vitro by allowing the study of the mechanisms implicated in the development and progression of this disease [19].

Despite the clear need for robust in vitro systems that mimic NAFLD, the development and employment of predictive assays are also essential, such as studying transcriptional expression, identifying potential protein and metabolite biomarkers, and running cytomic assays. The combination of results from well-validated in vitro models and new omic approaches will result in more predictive assays that can help to understand NAFLD and its progression, and that will also be useful during drug development.

Although clear progress in clarifying NAFLD etiopathogenesis and developing pharmacological treatments has been described, there are still many challenges to elucidate the mechanisms implicated in this disease and its progression, and to find new therapies. Therefore, basic preclinical research is essential. Here we review the major mechanisms of liver injury in NAFLD, and the advances made in the in vitro models and tools currently used for studying NAFLD, by focusing on employing iPSCs as a personalized medicine approach to study this disease.

## 2. Mechanisms of Liver Injury in NAFLD: Mitochondrial Dysfunction, Oxidative Stress, and Lipotoxicity

Uptake of non-esterified fatty acids (NEFA) from the diet, circulating NEFA released from lipolysis in adipose tissue, and de novo synthesis in the cytosol of hepatocytes are the major origins of NEFA present in the liver. The two major pathways to remove NEFA from hepatocytes are fatty acid oxidation in mitochondria (β-oxidation) and their export to blood as VLDL (very low-density lipoproteins). NEFA are stored mainly as triacylglycerides (TAG) in small intracellular fat droplets. TAG accumulation in hepatocytes is not necessarily a pathological condition, but is considered a protective mechanism to avoid the potential toxic effects induced by high NEFA levels, particularly saturated long-chain fatty acids [20].

A very close association between insulin resistance and NAFLD is well accepted [21]. Insulin resistance favors lipolysis in white adipose tissue, which leads to more NEFA being released to the liver. In the liver, hyperinsulinemia and/or insulin resistance result in hepatic lipid overaccumulation by down-regulating triglyceride export from the liver as VLDL, and by promoting the use of glucose (and fructose) for the de novo synthesis of lipids (lipogenesis) in the cytosol of hepatocytes [22,23]. Insulin-mediated effects on lipogenesis and lipid accumulation in the liver are strictly regulated by several nuclear receptors and transcription factors (e.g., SREBP1c, ChREBP, PPARs, and LXR) [24,25].

**Figure 1:**
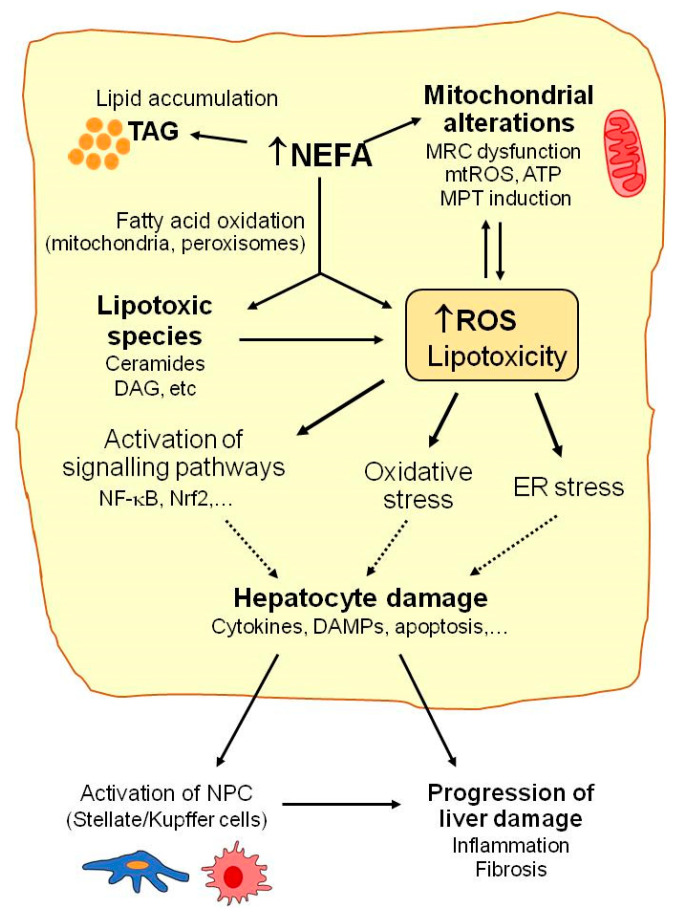
Mechanisms implicated in NAFLD. Many molecular pathways contribute to NAFLD development. When the liver’s capacity to control energy substrates is overwhelmed, toxic lipid species may accumulate. This can be associated with increased reactive oxygen species (ROS) production, mitochondrial injury (mitochondrial respiratory chain (MRC) alterations, increased mitochondrial ROS (mtROS), or mitochondrial membrane permeability transition (MPT) induction), hepatocellular stress, and liver injury. Excessively high non esterified fatty acid (NEFA) levels enhance triacylglycerides’ (TAG) accumulation and fatty acid oxidation in hepatocytes and favor ROS generation, which may contribute to alterations in mitochondrial function and metabolic energy homeostasis in hepatocytes. Accumulation of lipotoxic species (e.g., ceramides, diacylglycerol (DAG)) may aggravate mitochondrial dysfunction and contribute to ROS overproduction, oxidative stress, endoplasmic reticulum (ER) stress, and activation of the signaling pathways involved in hepatocyte injury, including the release of cytokines or damage-associated molecular patterns (DAMPs). Finally, activation of non-parenchymal cells (NPC) by oxidative stress or by the signals released by damaged hepatocytes may contribute to NAFLD progression.

Mitochondria play a key role in the homeostasis of lipid metabolism. Under normal physiological conditions, most NEFA enter mitochondria via carnitine palmitoyl transferase, where they are converted into acetyl-CoA by the β-oxidation pathway, with subsequent ATP generation via the tricarboxylic acid cycle and oxidative phosphorylation. With increased NEFA flux in hepatocytes, the upregulation of fatty acid oxidation in mitochondria is one of the first metabolic adaptations that is triggered in the liver to prevent fat overaccumulation [26,27]. Thus, increased electron flux to the mitochondrial respiratory chain favors the formation of reactive oxygen species (ROS), mainly superoxide anion, that can contribute to altering mitochondria function and integrity (reduced ATP synthesis, mitochondrial membrane depolarization, depletion of mitochondrial DNA, release of cytochrome c and pro-apoptotic factors, and impaired mitochondrial biogenesis) (Figure 1). In turn, these mitochondrial alterations can favor ROS overproduction and, thus, contribute to exacerbating mitochondrial damage and oxidative stress [28,29].

Moreover, with marked lipid metabolism dysregulation, which occurs in NAFLD, mitochondrial β-oxidation is insufficient to metabolize excess NEFAs, which accumulate in hepatocytes and may lead to more lipotoxic species forming (e.g., ceramides, diacylglycerols, lysophosphatidylcholines, or other lysophosphatidic acid derivatives, saturated fatty acids, and oxidized low-density lipoproteins) [20,30,31]. This excessive accumulation of fat and lipotoxic intermediates has deleterious effects on diverse cell organelles and functions, which are generally known as lipotoxicity (Figure 1). Mitochondrial dysfunction, endoplasmic reticulum (ER) stress, and the activation of signaling pathways related to inflammation and cell death are among the most prominent consequences of these deleterious effects [30].

Lipotoxic species such as ceramides can inhibit mitochondrial respiratory chain complexes and lead to ATP depletion, increased ROS production, the deregulation of the mitochondrial antioxidant defense system, and other mitochondrial disturbances and, thus, contribute to the aggravation of mitochondrial dysfunction and oxidative stress [28,32]. ER stress induced by lipotoxic agents has been related to ROS overproduction and exacerbated oxidative stress in NAFLD [33]. ER stress also contributes to reduced triglyceride synthesis and VLDL export, which increase intracellular fat accumulation in the liver. Lipotoxicity also up-regulates the signaling pathways (e.g., NF-κB, JNK, and NLRP3) that promote the release of the pro-inflammatory and pro-apoptotic mediators that actively participate in liver cell injury progression [28,34]. Not only hepatocytes but also non parenchymal liver cells (NPC, Kupffer, stellate, or sinusoidal endothelial cells) are susceptible to oxidative stress and lipotoxic species by playing important roles in NAFLD. The cytokines and DAMPs (damage-associated molecular patterns) released by damaged or dead hepatocytes may induce the activation of innate immune cells, the recruitment of macrophages, and the activation of Kupffer cells (KC), which trigger an inflammatory response in the liver. Oxidative species may also induce the activation of stellate cells and stimulate their collagen production to, thus, promote liver fibrosis [28]. KC and infiltrating monocyte-derived macrophages perform an important dual function in the development and progression of NAFLD [35]. They may release pro-inflammatory cytokines and promote liver injury or produce anti-inflammatory cytokines and limit disease progression [35]. It has been reported that exposure to saturated fatty acids (e.g., PA) favors the polarization of liver macrophages to the pro-inflammatory M1 phenotype, while unsaturated fatty acids (e.g., OA) promote KC differentiation to the anti-inflammatory M2 phenotype [36]. Further research is necessary to better understand the role of KC polarization in NAFLD and to clarify its potential utility as a future therapeutic target in NAFLD.

## 3. Liver Cell Models to Study NAFLD

**Figure 2:**
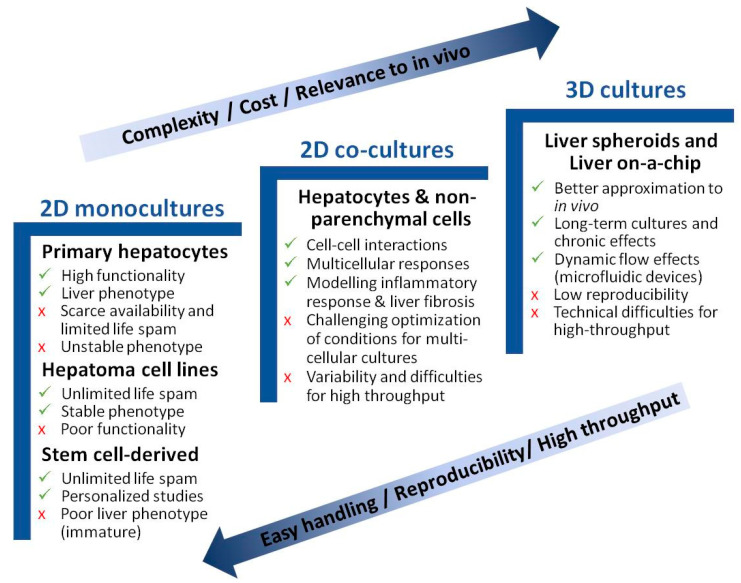
Advantages and disadvantages of in vitro models for studying NAFLD. Cell culture models are listed per increasing cost, longevity, and complexity. Simpler models are better suited for high-throughput applications.

The biological systems used for the in vitro study of NAFLD range from monolayer cell–cultures to more complex 3D cultures. The purpose is to recapitulate the biology of NAFLD and identify the specific pathways implicated in the pathogenesis of this disease, and to also find useful therapeutic targets for drug development. Apart from hepatocytes, different NPC, such as KC, liver endothelial cells (LEC), or hepatic stellate cells (HSC), may affect liver biology, and have been described to play an important role in NAFLD pathogenesis. In recent years, new in vitro models have been suggested for studying NAFLD pathogenesis. Yet, despite a majority of in vitro studies focusing on the biology of NAFLD, publications reporting compound testing have increased in the last three years [37]. Figure 2 schematically presents the major advantages and disadvantages of each described model. Table 1 summarizes the main in vitro models for studying NAFLD.

### 3.1. Monoculture Models

PHH are considered the closest in vitro model to the human liver. Hepatocytes are obtained by liver tissue digestion and retained in culture-specific liver functions, such as metabolic detoxification of foreign compounds, glycogen synthesis and storage, lipid metabolism, urea and albumin production, and the functional expression of membrane transport proteins [38]. Therefore, PHH have been proposed as an in vitro model for diverse hepatology fields, such as pharmacological and toxicological studies, and liver diseases modeling, including NAFLD. However, their widespread use is hindered by scarce liver tissue availability, high interdonor variability, and the short life and progressive loss of hepatocytes’ functionality during culture [39]. In contrast, immortalized hepatic cell lines offer an almost unlimited proliferation potential, high availability, easy handling, and a stable phenotype, and are widely used as alternative cell models to PHH. Their main disadvantage is the low expression of certain enzymes, mainly those involved in drug metabolism [40].

The most widespread approach to generate in vitro models of NAFLD is adding NEFA to cell culture medium. In particular, an NEFA mixture containing oleic and palmitic acids (OA and PA, at the 2:1 ratio) has been shown to induce lipid accumulation in both PHH and hepatoma cell lines (e.g., HepG2, Huh7, HepaRG, or LO2), which can be easily evidenced by Nile Red, BODIPY 493/503, or Oil Red O staining. Cells incubated with NEFA show the formation of cytosolic fat droplets, and their morphology resembles ballooned hepatocytes, which are typical of the steatosis process. The extent of lipid accumulation, mainly TAG, in PHH exposed to NEFA is similar to that observed in the liver of patients with steatosis [41]. Apart from lipid accumulation, induction of apoptosis, increased ER stress, and inhibition of protein synthesis have also been observed in NEFA-treated hepatocytes [41,42]. Cytotoxicity, increased ROS, and induction of oxidative stress, mitochondrial alterations, and production of inflammatory or fibrinogenic cytokines are other effects observed in NAFLD cell models [43,44,45,46]. The composition of the NEFA mixtures used to induce fat-overloaded cells can influence the observed effects. NEFA mixtures with a low proportion of saturated PA induced lipid levels display minor effects on cell viability and, thus, render an NAFLD cell model that mimics benign steatosis. In contrast, a high proportion of PA favors not only fat accumulation but also harmful cytotoxic and apoptotic effects [41].

In addition to NEFA, drugs or endocrine disruptors such as bisphenol A have been used to generate NAFLD cell models [47,48,49]. Drug-induced steatosis has been well-studied in vitro and associated with both increases in lipid accumulation and ROS formation in several liver cell models [50,51]. More detailed studies into HepG2 cells have provided a better understanding of the mechanisms associated with amiodarone, a well-known steatogenic compound that not only produces increased lipid accumulation but also changes in the expression of the genes related to lipid metabolism, such as *SREBP1c* and *DGAT1*, along with increased activation of ER-stress regulator IRE1α and induction of autophagy [52]. The steatogenic effects of drugs have also been assessed in Lo2 cells, where exposure to valproic acid increases cytoplasmic lipid levels and enhances oxidative stress, as reflected by the lower GSH level or the higher MDA and ROS levels. This increase has been linked with an increased expression of isoform 2E1 of cytochrome P450 (CYP), as valproic acid-induced ROS accumulation and hepatic steatosis were attenuated when CYP2E1 was inhibited using the CYP2E1 inhibitor or CYP2E1 CRISPR knockdown (CYP2E1-KD) [53].

A comparative study has revealed that HepaRG cells exhibit more sensitivity to drug-induced steatosis than HepG2 cells [54]. HepaRG cells are known to show a better expression of different drug-metabolizing enzymes and liver functions than other hepatoma cell lines, such as HepG2 [55]. Allard et al. recently studied the mechanisms by which 12 commonly used drugs produced steatosis in HepaRG, and observed different patterns [56]. For instance, a group of drugs that includes amiodarone and rifampicin caused steatosis by decreasing mitochondrial fatty acid oxidation, whereas another set of drugs (i.e., allopurinol, fluorouracil, and troglitazone) reduced the expression of the proteins involved in VLDL secretion, such as APOB and induced ER stress [56]. Regarding the antioxidant capacity of different cell lines, a comparative study of HepG2 and HepaRG cells showed a more significant decrease in GSH or increase in ROS in HepaRG than in HepG2 cells after treatment with compounds that are known to produce oxidative stress induction [57].

Single-cell in vitro models fail to accurately recapitulate the pathological mechanisms of liver diseases. Therefore, more physiologically relevant models such as cocultures or 3D systems have been developed.

### 3.2. Coculture Models

Although hepatocytes are the major cell type in the liver, they co-exist with other NPC, such as LEC, KC, and HSC, which play important roles in NAFLD development. Barbero-Becerra et al. studied the interaction between HuH7 and LX2 (a hepatic stellate cell line) and observed that NEFA exposure increases the α-smooth muscle actin (α-SMA) expression in LX2, regardless of the cell–cell contact with hepatocytes [58]. In a similar model, greater α-SMA production and an increase in extracellular matrix (ECM) components, such as collagen I, collagen II, collagen IV, fibronectin, and profibrotic proteins such as MMP-2 and MMP-9, have been reported and linked with higher oxidative stress [59]. Activation of cocultured HSC was related to up-regulation of antioxidant regulator Nrf2 in hepatocytes damaged by lipid accumulation, and Nrf2 was suggested as a potential therapeutic target to prevent or delay NASH progression. These studies evidenced the interplay between lipid accumulation in hepatocytes and HSC activation with a fibronogenic phenotype in co-culture models of NASH.

KC are resident macrophages in the liver responsible for detecting local damage and eliminating foreign substances. The coculture of KC and hepatocytes has been used to study not only the inflammatory process that accompanies NAFLD, but also possible therapeutic targets [60]. The coculture of mouse primary hepatocytes and KC in a transwell system incubated with PA has been used as a model of NASH and to study drug effects. The model confirmed the role of KC in NASH progression and elucidated different implicated molecular mechanisms [61]. Cocultured hepatocytes show higher sensitivity to the toxicity, due to some compounds with oxidative properties, than do hepatocyte monocultures [62]. This has been related to the generation of toxic metabolites that decrease GSH levels in hepatocytes and activate ROS formation by KC causing further GSH depletion.

### 3.3. 3D Models of NAFLD

By better understanding that 3D cultures more accurately reflect in vivo physiology, in the last few decades, many researchers have focused on developing and optimizing different 3D strategies to better preserve liver properties in vitro by mimicking the architecture and cell–cell interactions [63]. 3D models include scaffold-containing and scaffold-free systems. 

Cell spheroids are one of the most widely used scaffold-free strategies to generate 3D NAFLD models. This approach consists of generating cell aggregates by different techniques, such as hanging drop, low-adherence substrates, microwells, bioreactors, and magnetic levitation [64]. PHH spheroids maintain their viability and functionality up to 21 days and have been used to generate an NAFLD model after exposure to high levels of insulin, NEFA, and monosaccharides [65]. After a seven-day accumulation of intracellular lipids, the development of insulin resistance was detected in spheroids on day 14, as evidenced by the increased expression of *PCK1* and *PDK4* and by reduced GSK3β phosphorylation [65]. The spheroid methodology was followed to coculture HepG2 and LX-2 cells [66]. This system demonstrated that hepatic stellate LX-2 cells facilitate the compactness of spheroids, which confirms the role of HSC in matrix remodeling. Treatment with a mixture of NEFA brought about an increase in cytoplasmic lipids and a higher COL1A1 expression, and allowed the effects of anti-NASH drugs such as liraglutide or elafibranor to be studied [66]. Some 3D liver in vitro tissue models are commercially available, such as 3D InSightTM Human Liver Microtissues (InSphero). The system consists of PHH, HSCs, LEC, and KC, which are organized in microspheres. It has been used to model NASH with severe fibrosis by exposing microtissues to high PA concentrations, which results in the induction of a fibrotic and proinflammatory profile in tissue with increased expressions of IL-8 and collagen 1 and 3 [67]. This model has been described as particularly useful for mechanistic exploration of inflammation-associated processes that would be of special interest for studying the antioxidant response [68].

Native tissues comprise a 3D viscoelastic milieu, the ECM, which guides cells’ development, interactions, and homeostasis. Therefore, mimicking the natural ECM is a promising approach to gain a 3D structure for a cell culture that better represents what happens in vivo. For instance, the PHH cultured in a collagen-sandwich configuration retain higher CYP expression, greater metabolic activity, and a longer life span to, thus, delay the dedifferentiation process by up to 14 days in culture [69]. This system has also been used for the coculture of PHH, macrophages, and HSC in another system that incorporates both perfusion and hemodynamic shear forces [70]. Increases in cytoplasmic lipids and the synthesis of triglycerides, diglycerides, and cholesterol esters, accompanied by the expression of the genes associated with apoptotic and oxidative stress signals, have been observed after a 10-day exposure to high concentrations of glucose, insulin, and NEFA. Greater insulin resistance (increased *PCK1* expression and the generation of a proinflammatory profile) and a fibrotic profile (increased α-SMA, TGF-β, and steopontin) have also been reported [70].

Hydrogel-based systems are 3D hydrophilic polymeric networks that mimic ECM and allow the free diffusion of oxygen and nutrients. Duriez et al. developed a 3D NASH model by combining the culture of four cell types embedded in collagen hydrogel with exposure to glucose, NEFA, and TNF-α for 15 days. This model showed the accumulation of lipid droplets in the cytoplasm, the generation of a proinflammatory environment determined by increased IL6 and CCL2 expressions, and the induction of early fibrosis with the expressions of MMP2 and MMP9 [71].

### 3.4. Liver-on-a-Chip

The liver-on-a-chip methodology is based on combining different structures that recreate the conditions and dynamics of a small-scale liver. On these platforms, several polymeric chambers act as supports to generate cell cultures which are connected by channels that distribute microfluids with nutrients and oxygen [72]. This technology allows the generation of cell–cell interactions and a dynamic flow to increase cell survival and functionality. Liver-on-a-chip has emerged as a very powerful tool to not only study pathologies but to also preclinically study potential therapies [73,74]. The ability to connect different chambers also allows the combination of distinct tissues that may be important in disease development. Despite its advantages, this model is not widely used because of its high cost and considerable complexity.

The liver-on-a-chip technology has been utilized to model NAFLD by employing simple cell systems, such as HepG2 cells, and ranges to more complex ones that combine different cell types. The culture of HepG2 cells in an NEFA perfused device for 24 h and 48 h allows more gradual triglyceride deposition and increased cell survival which, thus, resemble chronic steatosis that develops in vivo [75]. This model confirmed that PA is more cytotoxic than OA and revealed a direct contribution of PA overload to induce oxidative stress (production of total ROS and superoxide) in liver cells [76].

Besides steatosis, inflammation and fibrosis are two important events in NAFLD development. The use of liver-on-a-chip that combines several liver cells (hepatocytes, HSC, LEC, and KC) and stimulation with NEFA and LPS results in increased cytoplasmic lipid accumulation, ballooned hepatocytes, and higher a-SMA, collagen 1A1, and TIMP-1 production, which are indicators of liver fibrosis [77]. The system recapitulates NASH endpoints and also allows testing of anti-NASH drugs, with a very high potential for further drug testing [77].

The organ-on-a-chip technology has been used to assess the role of other tissues in NAFLD development. Lee et al. constructed a gut–liver microfluidic chip and showed that most of the lipids that accumulated in hepatocytes while this disease developed were obtained from exogenous input and transport through the gut, and were key. Additionally, the efficacy of antisteatotic compounds, whose mechanism of action is based on altering lipid absorption at the intestinal level, has been evidenced [78]. More recently, the influence of lipolysis and insulin resistance on NAFLD development has been explored by a model of adipocyte and hepatocyte chambers. With this model, in a lipotoxic environment, adipocytes modulated adipokine secretion and lipolysis to increase hepatic steatosis [79].

## 4. Human Hepatocyte-like Cells Deriving from Pluripotent Stem Cells for NAFLD Modeling

Since their discovery, stem cells have been a proven potential tool for developmental biology, drug toxicity, or regenerative medicine thanks to their capability to proliferate and differentiate into somatic cells. The possibility of using stem cells to recreate some hallmarks of different diseases has also been explored in vitro, and has been demonstrated as a promising approach for disease modeling and a competent alternative to PHH and immortalized cell lines [82,83].

Human adult stem cells reside in specific niches inside tissues, and their multipotency allows them to differentiate into different tissue-specific somatic cells to contribute to tissue regeneration and renewal [84]. In fact, stem cells from different tissues, such as bone marrow, adipose tissue, or umbilical cord blood, have been differentiated into cells with the hepatic phenotype [85]. Human skin-derived precursors have been shown to differentiate into hepatic progenitor cells in vitro with suitable properties for drug hepatotoxicity studies [86], NAFLD modeling [87], and efficacy assessments of potential anti-NASH compounds [88,89]. Nonetheless, adult stem cells are rare, their isolation may prove difficult given tissue accessibility restrictions, and long times are required to extend the obtained cells in vitro to generate sufficient stock [90].

Human pluripotent stem cells (PSCs), such as embryonic stem cells (ESCs) and iPSCs, indefinitely proliferate and are capable of forming cells of all germ layers. PSCs can be differentiated into hepatocyte-like cells (HLCs) that are morphologically and functionally similar to PHH [91] and provide an unlimited source of HLCs that can be cultured longer before loss functionality.

**Figure 3:**
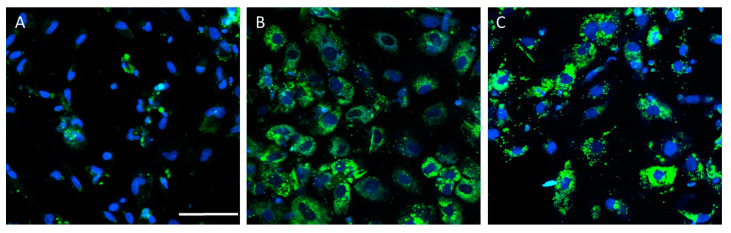
Drug-induced lipid accumulation in the hepatocyte-like cells (HLCs) derived from induced-pluripotent stem cells (iPSCs) from a healthy donor. Representative images of untreated HLCs (**A**), and HLCs treated with valproate (**B**) or amiodarone (**C**), are also shown. Nuclei were stained with Hoechst 33342 (blue) and neutral lipids with BODIPY 493 (green). Scale bar (50 µm) applies to all images.

PSC-derived HLCs have been used to generate in vitro models that recapitulate some NAFLD features (Table 2). Lipid accumulation has been accurately recreated in HLC-based models upon lipid overloading [19,92,93,94,95,96], high-energy substrate induction [97], or steatogenic drug exposure [98]. Figure 3 depicts a representative example of increased lipid accumulation induced by drugs in an NAFLD model of HLCs deriving from iPSCs.

Other NAFLD hallmarks, such as mitochondrial dysfunction and oxidative stress, have been recreated in different HLC models [95,97]. Some cell stressors, such as ER stressor thapsigargin, are useful for potentiating the steatosis phenotype upon NEFA overload in iPSC-derived HLCs, which confirms the link between ER homeostasis and lipid metabolism. This exacerbated phenotype has allowed the effects of the therapeutic drug tauroursodeoxycholic acid and experimental compound obeticholic acid to be evaluated, which reduced the steatosic phenotype in iPSC-derived-HLCs [94]. This scenario suggests that HLC-based NAFLD modeling is a promising tool for testing potential anti-NASH compounds. HLCs have also been described as a potential platform for assessing drug-induced steatosis and as a similar cell model to HepG2 [98].

One of the major advantages of iPSCs is that they can be obtained by a well-standardized reprogramming process using somatic cells, which can be obtained by minimal invasive methods (blood or urine collection). The iPSCs from patient somatic cells can be employed to generate HLCs, which maintain the patient-specific genotype and metabolic variations [99,100] to allow the study of the interindividual variability associated with disease development and progression, as in NAFLD [83]. In fact, the iPSC-derived HLCs from NAFLD patients have been developed to obtain a potential NAFLD model to study the idiosyncratic nature of this disease [101,102]. An HLC-derived NAFLD cell model from different NAFLD patients has revealed some differences in the steatotic phenotype among donors, including differences in drug response. By analyzing all the transcriptome data from a single donor, a different response to AdipoRon (an adiponectin-like small molecule that can revert the steatotic phenotype) treatment has been observed, which could be partly due to variations in donors’ individual genetic backgrounds [103].

Specific genetic alterations related to increased NAFLD susceptibility can be modeled with HLCs derived from NAFLD patients. For instance, mutations in patatin-like phospholipase domain-containing protein 3 (*PNPLA3*), such as variant I148M (responsible for loss of enzymatic activity [104]), have been successfully recreated in iPSC-derived HLCs by the CRISPR/Cas9 technology for either knockout *PNPLA3* or the knockin I148M variant [96]. *PNPLA3*I148M–HLCs exhibit a similar lipidomic profile to liver biopsies from donors with the I148M variant and show more susceptibility to other toxicity types, due to the down-regulation in the detoxification pathways related to variant I148M [96].

Nevertheless, 2D monocellular HLC models failed to recapitulate some NAFLD hallmarks because the cytoarchitecture was lacking, which mimics liver parenchyma and critical hepatocyte–NPC interactions [93]. Liver organoids have been developed with PSCs, adult stem cells, or fetal/adult liver cells. Under specific culture conditions (including growth factors and cytokines), stem cells differentiate and progress, like liver development, to generate adult somatic cells in a 3D environment and to give organoids that mimic structural and functional liver parameters [105]. These hepatic organoids not only recreate NAFLD hallmarks (e.g., lipid accumulation, increased lipid peroxidation, and higher ROS levels), but also show bile canaliculi network disruption upon lipid induction [95], as described in NAFLD-patient samples [106]. Lipid droplet accumulation in HLCs has been observed in the organoids formed by HLCs from NAFLD patient-derived iPSCs and NPC lineages (macrophages, mesenchymal stem cells, and endothelial cells) when exposed to an NEFA mixture. Interestingly, Gurevich et al. noted how these NAFLD patient-derived HLCs organoids displayed spontaneous lipid accumulation without lipid overloading [93].

The bioengineered tissue formed by iPSC-derived HLCs with an inducible knockdown expression of deacetylase sirtuin-1 (*SIRT1*), cultured in combination with macrophages, has been reported to mimic the pro-inflammatory phenotype upon lipid overloading and revealed the major implication of sirt1 in de novo lipogenesis and β-oxidation regulation, along with their pivotal role in NAFLD development and progression [92]. Thus, 3D models are powerful tools to elucidate the mechanisms underlying NAFLD development with a view to study cell interactions in the progression of this disease. However, these cell culture models are still expensive and need further technological improvements.

HLCs are a proven competent tool for mimicking several NAFLD hallmarks in vitro by studying the cellular mechanisms implicated in the disease and their progression, and the genetic alterations related to NAFLD development, by recreating the NAFLD patient-specific genotype and learning about the genetic predisposition to NAFLD and its idiosyncrasy. This personalized in vitro approach would better reflect specific pathobiological features of disease among NAFLD patients, such as metabolic status or sex differences. Table 2 summarizes the different PSC-cell models used to study NAFLD. Three-dimensional cultures of HLCs more closely recreate the liver’s cytoarchitecture (including the bile network) than 2D cultures, while 3D cocultures with NPCs have revealed the possibility of more accurately mimicking other NAFLD hallmarks, such as the proinflammatory phenotype. Nevertheless, more studies are required, and other approaches should be considered, such as including other NPCs in HLCs cocultures, like HSCs, which play an important role in fibrotic NAFLD progression to NASH [58]. Disease modeling based on HLCs has to deal with other drawbacks, such as the inability to obtain a fully mature phenotype in HLCs, because they exhibit characteristics of immature hepatocytes [91,99,107]. Therefore, differentiation protocols are constantly improving to obtain mature HLCs, which have become the best starting point for disease modeling [108]. Although stem cell-based NAFLD modeling is a promising tool in the research and clinical fields, it cannot be denied that further research is needed to standardize culture conditions.

## 5. New Approaches and Tools for the In Vitro Assessment of NAFLD

NAFLD is characterized by alterations in a wide range of molecules, such as proteins, NEFA and other lipids, and metabolites [109], and is also related to changes in expression patterns [110]. Therefore, a wide spectrum of techniques (staining lipids with specific dyes, coupled-reaction assays for metabolites, enzyme activity measurements, analysis of protein expression by immunochemistry or Western blot analyses, or analysis of mRNA by RT-PCR) has been routinely followed to identify and analyze cellular, biochemical, and functional changes in NAFLD models. By applying these traditional methodologies, only a single parameter (or a few parameters) is evaluated in each assay, which is time-consuming and seriously limits the full characterization of NAFLD-related events. In contrast, omics-based technologies allow the simultaneous analysis of many parameters in the same cell system and offer the possibility of performing more comprehensive mechanistic studies about the global events involved in NAFLD.

Many omics studies have been performed to analyze patients’ samples (plasma, liver tissue, faeces) or samples from in vivo experimental models of NAFLD, to find new biomarkers for the prognosis, diagnosis, or monitoring of the disease [111,112,113,114,115]. Omics studies have also been applied to NAFLD in vitro models as novel high-throughput strategies to search and identify new NAFLD biomarkers or therapeutic targets, and to test new molecules as potential drugs to treat NAFLD (Table 3). In many studies, the in vitro results obtained in NAFLD cell models have been validated with, or compared to, data from in vivo serum-based studies.

Transcriptomics is probably the most widely used omics for the in vitro study of NAFLD. Both microarray and RNA sequencing (RNASeq) technologies have been followed to identify and quantify RNA expression profiles in in vitro NAFLD models (Table 3). For instance, untargeted microarray analyses in steatotic and non steatotic Huh7 cells [116] have revealed 88 differentially expressed genes, including cytokine CXCL10, which was previously identified by in vivo studies as a powerful biomarker for NASH [127,128]. A transcriptomic analysis in an NAFLD model in PSC-derived HLCs has identified a steatotic profile, which includes numerous genes related to the PPAR pathway [19], as previously demonstrated in vivo [129]. The transcriptomics analysis has also been applied to evaluate drug candidates to treat NAFLD in vitro [88,100]. Some studies have also focused on the RNA-based regulatory mechanisms that underlie NAFLD, because transcriptomics has allowed the identification and quantification of non-coding RNAs, such as microRNAs (miRNAs) [19] or long non-coding RNAs (lncRNAs) [119,120].

Proteomics, metabolomics, and lipidomics (the branch of metabolomics that centers on studying lipidic perturbations that is especially relevant in steatosis studies) have also been useful in the in vitro study of NAFLD. The ever-growing use of these three analytical tools for research purposes has been driven by the recent methodological advances made in chromatography, either gas (GC) or liquid (LC), and mass spectrometry (MS). The analysis of the proteomic, metabolomic, and lipidomic profiles in in vitro NAFLD models may provide additional global insights into the mechanisms involved in NAFLD to identify new specific biomarkers for the clinical diagnosis of NAFLD or to discover novel drugs to treat NAFLD patients. By way of example, an untargeted proteomics study in an octanoate-based model of steatosis with C3A hepatoblastoma cells has demonstrated that lipid metabolism-related proteins, such as serum albumin, Perilipin-2, APOAI, AKR1C1, or FABP1, are the most altered proteins [121]. Metabolomics studies with a model of steatosis using OA/PA-treated HepaRG cells have shown significant changes in lipids, glutathione, carnitine, and tricarboxilic acid cycle intermediates, and metabolites related to oxidative stress, energy metabolism, and insulin resistance [43]. Similar results have been reported by Cuykx et al. in a metabolomics study with a valproate-induced HepaRG model of steatosis [123]. They found perturbations in the levels of lipids, such as ceramides, tryglicerides, and carnitine, among others. An untargeted lipidomics study with an OA/PA-treated HepG2 model of steatosis has demonstrated that levels of phospholipids, triglycerides, ceramides, and sphingomyelins alter under steatogenic conditions [124]. All these studies sustain the suitability of proteomics, metabolomics, and lipidomics studies for assessing NAFLD in vitro.

The application of combined strategies with two omics technologies or more will lead to a more exhaustive and global NAFLD characterization and will contribute to a better understanding of all the involved mechanisms. Along these lines, a few reports illustrate the application of this integrative strategy to study NAFLD in diverse cell liver models, such as PHH, hepatoma cell lines, or HLCs [70,97,125,126]. However, the interpretation and integration of data from multiple omics are difficult because alterations in gene or protein expression and metabolite levels occur on different time scales. 

Finally, cytomics, the comprehensive, structural, and functional study of the cytome at a level of individual cells, has been proposed as a multiparametric tool to examine the complex and dynamics biology of in vitro cell models. Cytomics is based on using multiplexed cell staining assays to analyze alterations in cell structures or functions. Flow cytometry is the main technique followed in cytomics studies, but some microscopy-based technologies such as high-content screening (HCS) have also been applied to study NAFLD in vitro. For instance, different fluorescent probes can be used to stain and quantify lipids [126]. Flow cytometry has been applied and/or HCS assays have been run to study drug-induced steatosis in HepG2, HepaRG, or Upcyte cells [50,51,54,81]. This technique also allows the combination of several fluorescent probes to identify steatogenic drugs with high sensitivity and to analyze the key mitochondrial alterations and oxidative stress associated with increased intracellular lipid levels [130].

## 6. Conclusions

NAFLD is a complex disease that can be triggered by a combination of different genetic, metabolic, immunological, and dietary factors. Moreover, there is compelling evidence for sex differences and the effect of reproductive status on NAFLD that remain largely unexplored [9]. Experimental methodologies in NAFLD should allow us to understand the pathogenesis of this disease, to evaluate the response to drugs, and to assess differential responses and pathogenesis due to gender. In the past few years, the diversity and complexity of cellular models used to study NAFLD have increased. However, these in vitro test systems still have many limitations and need further technological developments to provide a more in-depth mechanistic understanding of the disease and the implicated therapeutic mechanisms. The use of iPSCs deriving from patients and differentiated into HLCs may recapitulate many human NAFLD features and allow the effects of one drug, or more, on the key relevant pathways for disease progression to be assessed. The use of IPSCs deriving from donors with different NAFLD disease grades provides a new valuable tool to study whether differential responses to treatments are partially due to variations in individual genetic backgrounds. As traditional 2D cultures do not recapitulate native 3D spatial organization and intercellular interactions, new in vitro models (3D organoids, hydrogels, or liver-on-a-chip systems) attempt to mimic the real microenvironment of liver cells to provide more valuable and predictive models. Finally, the application of readout technologies that offer an exhaustive analysis of alterations at different levels by transcriptomics, metabolomics, or proteomics provides powerful mechanistic information that can even be applied to clinical practice, after an appropriate validation process. Although there is still much work to be done, the combination of new in vitro models that better recapitulate NAFLD and the use of new methodological approaches are expected to help us understand the disease and to extend these preclinical findings to humans.

## Figures and Tables

**Table 1:** Cellular models to study NAFLD in vitro.

Cellular System	NAFLD Induction	NAFLD Outcome	Observations	Ref.
2D Monocultures				
PHH	NEFA	Steatosis, ER stress	Lipid accumulation, apoptosis, activation of autophagy (IRE1a), and lipid metabolism (ATF6a)	[41,42]
HuH7	NEFA	Steatosis, oxidative stress, inflammation	Lipid accumulation, apoptosis, expression *IL-6, IL-8, TNFα*, increased ROS, *TGFB-1, TGFB -2, VEGF2*	[44]
HepG2	NEFA	Steatosis, oxidative stress	Lipid accumulation, increased ROS, mitochondria changes (ATP levels, mitofusin-2 expression), impaired cholesterol efflux, and ABCA1 expression	[45,46]
	Endocrine disruptors	Steatosis, oxidative stress, lipoperoxidation, blocking autophagy	Lipid accumulation, TBARS expression, accumulation of autophagosomes, decreased SQSTM1/p62 degradation	[48,49]
	Drugs	Steatosis, oxidative stress, blocking autophagy	Lipid accumulation, increased lipogenesis (*SREBP1c*) and triglyceride formation (*DGAT1*), ROS generation, decreased SQSTM1/p62 degradation	[50,51,52]
L02	NEFA	Steatosis	Lipid accumulation, up-regulation of relevant cholesterol synthesis genes	[80]
	Valproic acid	Steatosis, oxidative stress	Lipid accumulation, decreased GSH level, increased MDA and ROS levels	[53]
HepaRG	Drugs	Steatosis	Decreased β oxidation, expression of enzymes involved in lipogenesis or decreased proteins involved in VLDL secretion	[56]
Upcytes	Drugs	Steatosis, oxidative stress	Lipid accumulation, decreased *FOXA1* expression, increased ROS	[81]
2D cocultures				
HuH7 and LX2	NEFA	Steatosis, HSCs activation	Lipid accumulation, α-SMA expression	[58]
AML12 and HSC	NEFA	Steatosis, oxidative stress, HSCs activation	Lipid accumulation, ROS induction, decreased CAT, SOD, and GPx, expression of profibrotic molecules (α-SMA, Col I, MMP-2, MMP-9, fibronectin)	[59]
PHH and KC	NEFA	Steatosis, inflammation	Lipid accumulation, expression of lipogenesis enzymes (FASN, SREBP1c),expression of TNFα, IL-1β, IL6	[61]
3D models				
PHH	NEFA and insulin	Steatosis, insulin resistance	Lipid accumulation, increased expression of *PCK1* and *PDK4,* and reduced GSK3β phosphorylation	[65]
HepG2 and LX2	NEFA	Steatosis, fibrosis	Lipid accumulation, Col1A1 expression	[66]
3D InSight^TM^	NEFA	Fibrosis, inflammation	Expression of collagen genes, fibronectin, *α-SMA*, *IL-8* expression	[67]
PHH, HSC and macrophages	NEFA, insulin and glucose	Steatosis, insulin resistance, inflammation, fibrosis	Lipid accumulation, increased TAG, DAG and CE and PCK1 expression, reduced Akt phosphorylation, expression of IL-8, IL-6, and CXCL10, expression of TGF-β, OPN and α-SMA	[70]
PHH, HSC, LEC and KC	NEFA, TNFα and glucose	Steatosis, inflammation, fibrosis	Lipid accumulation, expression of IL-6, CXCL8, CXCL10, expression of MMP2 and MMP9	[71]
Liver-on-a-chip				
HepG2	NEFA	Steatosis	Lipid accumulation, increased TAG	[75]
PHH, HSC, KC, and LSEC	NEFA and LPS	Steatosis, liver injury, fibrosis, inflammation	Lipid accumulation, ballooned hepatocytes, increased Caspase 3, expression of α-sma, col1a, timp-1, tgf-β and opn, increases in tnf-α, mip1a, and mcp1	[77]
HepG2 and gut cells	NEFA	Steatosis	Lipid accumulation	[78]

**Table 2:** Pluripotent stem cell-derived cell models to study NAFLD.

Cell Model	HLCs Characterization	NAFLD Induction and Model Observations	Ref.
ESC line H1 and iPSCs from healthy donors	Polygonal shape, alb^+^, ecad^+^, hnf-4α^+^, urea synthesis, CYP3A4/3A5/3A7 activities, release of indocyanine dye	NEFA for 48 h. Lipid accumulation. Up-regulation of lipid metabolism regulators *PPARα* and *PLIN2*. Down-regulation of certain microRNAs (microRNA hsa-miR-122 and hsa-miR-106b).	[19]
HLCs from NAFLD donors (distinct grades of steatosis)	Polygonal shape, alb^+^, afp^+^, ecad^+^, hnf-4α^+^, a1at^+^, and ttr; low *CYP3A4* expression	NEFA for several days. Lipid accumulation with a donor-specific pattern. Increased *PLIN2* expression with differences between donors. Low expression of the genes associated with FGF21 signaling, lipid and cholesterol biosynthesis, and gluconeogenesis, with a low expression of *CPT1A* in high steatosis lines. AdipoRon effect on metabolism, transport, and signaling pathways.	[103]
iPSCs-Hep from female healthy donors	Polygonal shape, alb^+^, afp^+^, pou5f1^+^, hnf-4α^+^, expression of phase I, II, and III enzymes	NEFA and TAG for 18 h. PA dose-dependent lipid accumulation. Lipid accumulation exacerbated by TAG treatment, which induces ER stress by UPR dysregulation. Up-regulation of the genes related in fat storage in lipid droplets. Down-regulation of β-oxidation genes (*ACADM*) with FA-TAG treatment. Reduced TAG accumulation after inhibition of ER stress by therapeutic molecules tauroursodeoxycholic acid or obeticholic acid.	[94]
iPSC lines FSPS13B and A1ATDR/R obtained by CRISPR/Cas9 technology	Polygonal shape in HLCs alb^+^, a1at^+^, hnf-4α^+^ Detected CYP3A4 activity	NEFA for 7 days. HLCs-*PNPLA3^KO^* and HLC-*PNPLA3^I148M^* do not activate UPR markers (*BIP, GADD34, CHOP,* and *PERK*) with PA treatment, which indicates lipid-associated ER stress alterations. Lower levels of several β-oxidation gene expressions. Down-regulation of the genes implicated in drug detoxification, glucose metabolism and cell stress, but sensitivity to insulin remaining. HLCs-*PNPLA3^I148M^* recapitulate the main *PNPLA3*-associated NAFLD features.	[96]
Female H9 ESCs	Polygonal shape, alb^+^, hnf-4α^+^ Detected secreted albumin and CYP1A2 and CYP3A4 activities	Lactate, pyruvate and octanoate for 48 h or 96 h. Increased lipid accumulation. Tricarboxylic acid cycle dysregulation and altered expression of related enzymes. Alteration of β-oxidation and oxidative phosphorylation. Up-regulation of lipid vesicle transport proteins *PLIN1, PLIN2 or APOA4*, and gluconeogenesis genes. Transcriptional dysregulation in insulin resistance mediators. Induction of oxidative stress.	[97]
iPSCs from NASH and healthy patients	Polygonal shape with bile canaliculi formation. Hepatic markers (aat^+^, alb^+^), mRNA expressions of *CYP3A4, CYP3A7, SERPINA, ASGR1,* and *ALB*	NEFA for 24 h. Increased dose-dependent intracellular lipid accumulation. NAFLD patient derived HLCs organoids spontaneously accumulate lipids. HLCs were able to successfully integrate into 3D liver organoids with macrophages, MSCs and endothelial cells allowed NAFLD to be more accurately modeled.	[93]
3D coculture (HLCs and other cells in decellularized rat liver)	Polygonal shape, alb^+^, hnf-4α^+^ mRNA expressions of the enzymes, transporters, hepatic nuclear receptors, and transcription factors involved in liver metabolism	Linoleic acid and OA in the presence of DOX for 2–4 days. Increased lipid accumulation, lipid peroxidation levels, and total cholesterol levels. Increased mRNA expression of the genes implicated in de novo lipogenesis (*SREBP1c*). Decreased mRNA expression of β-oxidation key modulators (*PPARα* and *PGC1α*). Bioengineered tissue exhibits steatosis and expresses pro-inflammatory markers.	[92]
3D organoids (HLCs and CLCs deriving from PSCs)	alb^+^ (HLCs), ck7^+^ (CLCs), mRNA expression of proteins related to bile acid synthesis/secretion, cholesterol, fat and carbohydrate metabolism, drug detoxification, and hepatic-specific transcription factors	NEFA exposure. Increased lipid accumulation, ROS levels, and lipid peroxidation. Increased expression of the genes related to lipid and carbohydrate metabolism. Bile canaliculi network disruption.	[95]

**Table 3:** Omics approaches for the in vitro modeling of NAFLD.

Omics/ Technique	Cell Model (NAFLD Induction)	Observations	Ref.
Transcriptomics			
Microarray	HuH7 (NEFA)	Increased expression of interferon-stimulated genes and NF-kB-dependent pro-inflammatory genes	[116]
	HLCs and HepG2 (NEFA)	Increase in the PPAR pathway genes and Perilipin-2	[19]
	HepG2 and HSCs (NEFA)	Up-regulation in the ER-stress pathway genes	[117]
	PHH, HepG2, and HuH7 (NEFA and TNFα)	Comparison of different test systems. Changes in the genes linked with lipid droplet formation and metabolism (i.e., *HSDL2*)	[118]
	HLCs treated (NEFA, TNFα, IL1β, glucose, Insulin, and TGF1β)	Testing the anti-NASH compound (elafibranor). Gene expression profile and inflammatory markers of NASH	[88]
RNASeq	3D cocultures of PHH, HSCs, KC, and LSEC (NEFA, glucose, and TNFα)	Time course effects (3, 8, 10 days). 468 differentially expressed genes related to immune cell adhesion and inflammatory pathways	[71]
	HepG2 (NEFA & TNFα)	Evaluation of lncRNAs profiling in a model of steatohepatitis	[119]
	HepG2 (NEFA)	Differential expression of lncRNAs in untreated and steatotic cells with and without treatment with exendin-4	[120]
Proteomics			
HPLC-MS	C3A cells (lactate, pyruvate, octanoate, and ammonia)	104 differentially expressed proteins as indicators of enhanced protein synthesis accompanied by a down-regulation of histones	[121]
NLC-MS	HepG2 (NEFA, and menadione)	Identification of the differentially expressed carbonylated proteins (i.e., *ATP5A*) in NASH	[122]
Metabolomics			
GC-MS, UHPLC-MS	HepaRG (NEFA)	Global metabolomic analysis. Increased levels of branched chain amino acids and TCA cycle intermediates. Reduced carnitine and GSH levels	[43]
HPLC-MS	HepaRG (valproic acid)	Exposure to different concentrations and exposure times of VPA resulted in the identification of a typical steatotic profile: decreased carnitine, SAMe, and PEs in combination with the up-regulation of neutral heavy chain lipids	[123]
HPLC-MS	3D PHH spheroids (NEFA, insulin, glucose, and fructose)	Identification of the metabolites up-regulated in steatosis after 7 and 21 days of treatment. Study of the response to drug treatments	[65]
HPLC-MS	HepG2 (NEFA and drugs)	Identification of phospholipidosis- and steatosis-specific metabolites (NEFA, acylcarnitines, monoacylglycerides, diacylglycerides, and TAG) after incubation with phospholipidogenic and steatogenic compounds	[124]
Combined strategy			
Microarray & HPLC-MS	C3A (NEFA, lactate, pyruvate, octanoate, & ammonia)	Proteogenomics analysis revealed three candidate genes (fibrinogen α, β and γ chains) and their relation to cardiovascular risk associated with NAFLD patients	[125]
RNASeq & GC-MS (lipidomics)	HuH7 and PHH (NEFA, fructose, & insulin)	Studying the effects of media nutritional substrates on intracellular lipid accumulation by means of lipidomics (altered glucose metabolism, FA oxidation, and lipoprotein secretion) and transcriptomics	[126]
Microarray & UHPLC-MS	HLCs (lactate, pyruvate, & octanoate)	HLCs treated with lactate, pyruvate, and octanoate recapitulate the transcriptional and metabolic dysregulation of NAFLD The epigenomic analysis revealed the retained expression of TET enzymes and 5hmC	[97]
RNASeq & UHPLC-MS (lipidomics)	HPP, HSCs, and hMP (NEFA, glucose, & insulin)	The model recapitulated lipotoxic stress with a similar therapeutic drug response of NASH patients. High ATP and β-oxidation levels	[70]

## Abbreviations

3D: three dimensional; α-SMA: α-smooth muscle actin; CYP cytochrome P450; DAMPs: damage-associated molecular patterns; ECM: extracellular matrix; ER: endoplasmic reticulum; ESC: embryonic stem cells; MRI: magnetic resonance imaging; NEFA: non esterified fatty acids; GC: gas chromatography; HCS: high-content screening; HLCs: hepatocyte-like cells; HSC: hepatic stellate cells; iPSCs: induced-pluripotent stem cells; KC: Kupffer cells; LC: liquid chromatography; LEC: liver endothelial cells; MS: mass spectrometry; NAFLD: nonalcoholic fatty liver disease; NASH: nonalcoholic steatohepatitis; NPC: non parenchymal cells; OA: oleic acid; PA: palmitic acid; PHH: primary human hepatocytes; PSC: pluripotent stem cells; ROS: reactive oxygen species; SIRT1: deacetylase sirtuin-1; TAG: triacylglyceride; VLDL: very low-density lipoproteins.
